# A Narrative Review of Multifactorial Determinants of Childhood Eating Behaviors: Insights and Interventions Using the Social Ecological Model

**DOI:** 10.3390/children12030388

**Published:** 2025-03-20

**Authors:** Qutaibah Oudat, Sarah E. Messiah, Alia Dawlat Ghoneum, Anas Okour

**Affiliations:** 1 Department of Population Health, College of Nursing, University of Cincinnati, Cincinnati, OH 45221, USA; okourao@mail.uc.edu; 2 Peter O’Donnell Jr. School of Public Health, University of Texas Southwestern Medical Center, Dallas, TX 75390, USA; sarah.messiah@utsouthwestern.edu; 3 Department of Family Medicine, East Carolina University, 101 Heart Drive, Greenville, NC 27834, USA; ghoneuma23@ecu.edu

**Keywords:** childhood eating behaviors, social ecological model, family-centered interventions, school nutrition, health literacy

## Abstract

**Background/Objectives**: Childhood eating behaviors result from a complex interplay of familial, social, and environmental factors, influenced by socioeconomic and cultural contexts. These behaviors impact dietary habits, nutritional status, and long-term health. Using the Social Ecological Model (SEM), this narrative review synthesizes evidence on key determinants of childhood eating behaviors and proposes a framework for multi-level interventions. **Methods**: A structured literature search was conducted across PubMed, PsycINFO, and CINAHL, focusing on studies published between January 2014 and September 2024. Keywords related to childhood eating behaviors, familial determinants, and social influences were used to identify relevant studies. Inclusion criteria centered on empirical research examining how social and familial factors impact childhood eating behaviors within the SEM framework. **Results**: The review highlights critical determinants, including parental feeding practices, home food environments, peer influences, screen time, school meal programs, and socioeconomic disparities. These factors interact across multiple levels, emphasizing the importance of holistic interventions that target both individual behaviors and broader systemic influences. **Conclusions**: Addressing childhood eating behaviors requires a multi-level approach that integrates caregiver education, peer-led interventions, improved food environments, and supportive policies. Healthcare providers and policymakers play a crucial role in implementing strategies that foster healthier dietary behaviors and mitigate childhood obesity risks.

## 1. Introduction

Childhood eating behaviors are shaped by a complex interplay of social and familial determinants that collectively influence dietary habits, nutritional status, and long-term health outcomes. Early childhood is a critical period for establishing lifelong eating patterns and preferences, as children are highly impressionable and significantly affected by their immediate environment, particularly their family and household context [1]. Caregivers play a fundamental role in modeling eating behaviors, setting dietary norms, and providing the structure necessary for healthy eating habits [2,3,4]. Beyond the household, broader social factors, including cultural norms, socioeconomic status, and peer influences, further contribute to shaping children’s dietary behaviors [5,6]. Recent studies highlight the intricate nature of these interactions, demonstrating how caregivers’ attitudes and feeding practices directly impact children’s food choices and consumption patterns [7,8,9]. For example, positive parental modeling of healthy eating, authoritative feeding styles, and a supportive home food environment are linked to higher dietary quality in children [10,11]. Conversely, restrictive or pressure-based feeding practices, combined with easy access to unhealthy food options, have been associated with negative eating behaviors such as overeating, picky eating, and a preference for unhealthy foods [12,13].

However, children’s eating habits are not solely shaped within the family unit. As they grow older and gain more autonomy, schools can play a crucial role in dietary socialization by providing formal nutrition education through health workshops, and school curricula, equipping children with knowledge about healthy eating [14]. Additionally, school meal programs, cafeteria environments, and the availability of food in school stores or vending machines play a crucial role in shaping children’s dietary patterns [15,16,17]. These settings can either promote nutrient-rich options or increase exposure to less healthy alternatives. Beyond formal education, peer influence significantly shapes children’s eating behaviors. The desire for social acceptance and belonging often leads them to adopt dietary habits that align with their peer group, reinforcing either healthy choices or unhealthy patterns based on prevailing social norms [18]. Peer modeling further influences food preferences, affecting children’s willingness to try new foods or avoid certain food groups [19,20]. In addition to schools and peer dynamics, factors like screen time, mass media, and the Internet have become powerful drivers of children’s dietary behaviors. Food advertisements, social media influencers, and digital marketing play a significant role in shaping food preferences, consumption patterns, and brand loyalty [21]. Studies indicate that frequent exposure to digital marketing of unhealthy foods is associated with a higher preference for energy-dense, nutrient-poor snacks and beverages, contributing to poor diet quality [21,22]. Conversely, online educational campaigns, interactive nutrition apps, and digital interventions can offer opportunities to promote healthier eating behaviors and counteract negative influences [23,24].

The influence of familial and social determinants is further moderated by cultural and socioeconomic contexts [25]. Socioeconomic disparities contribute to variations in food availability, accessibility, and quality, which in turn affect children’s eating behaviors. Cultural traditions also shape dietary preferences, with some cultures emphasizing communal eating practices while others prioritize specific food groups [26,27]. The complex interplay between these determinants underscores the need for multi-level, culturally competent interventions that promote healthy eating habits among children.

**Figure 1:**
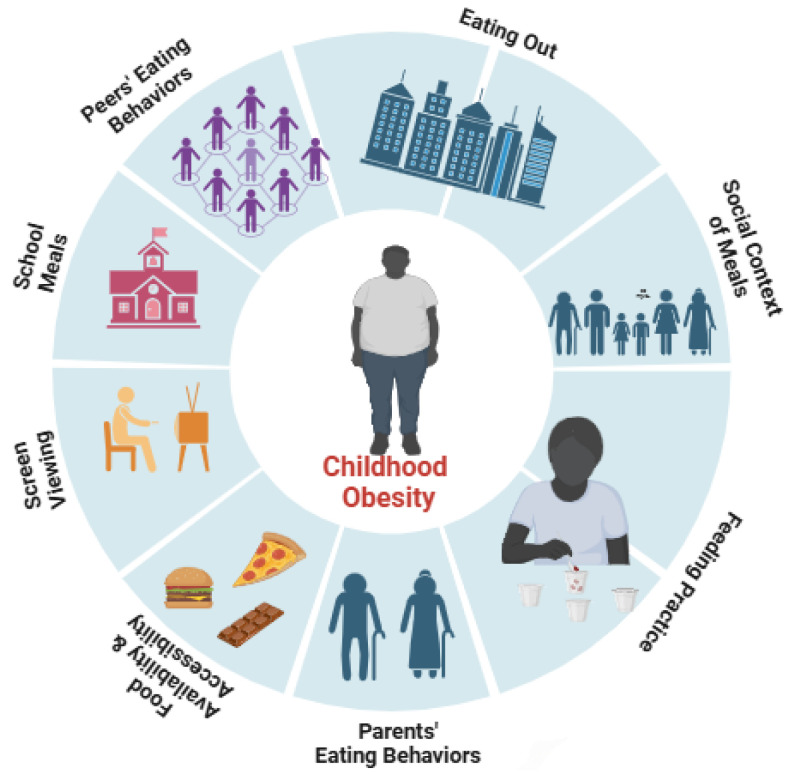
Multi-determinants affecting the eating behaviors of children.

A structured, theoretical framework is essential for comprehensively understanding how familial and social determinants shape childhood eating behaviors, requires a structured framework grounded in key theoretical constructs. While previous studies like [3,26,28] have extensively examined individual determinants of childhood eating behaviors, many studies have focused on isolated factors rather than considering the interconnected, multi-level influences that shape dietary habits. Additionally, prior studies have often lacked an integration of recent shifts in food environments, digital media exposure, and evolving family dynamics, all of which influence eating behaviors in unique ways. Furthermore, there is a scarcity of updated reviews that comprehensively synthesize emerging evidence within the Social Ecological Model (SEM) framework. This narrative review aims to bridge this gap by providing a current, multi-level synthesis of the factors influencing childhood eating behaviors and proposing actionable intervention strategies. This review was guided by the SEM (Figure 1) to provide a comprehensive synthesis of existing research on familial and social determinants impacting the eating behaviors of children, particularly those under 12 years old. The SEM offers a valuable lens through which the multi-layered interactions between individual factors interpersonal relationships, community environments, and policy influences can be examined. By utilizing the SEM framework, this review not only identifies determinants but also emphasizes the need for multi-level interventions that address these complex influences collectively, offering a roadmap for more targeted and effective strategies in promoting healthier eating behaviors among children.

## 2. Materials and Methods

This narrative review was structured using the SEM as a guiding framework to explore the diverse and multifactorial determinants of childhood eating behaviors. To ensure a comprehensive synthesis of relevant literature, a structured search was conducted across PubMed, PsycINFO, and CINAHL (via EBSCOhost), focusing on studies published between January 2014 and September 2024. Recognizing the evolving nature of research in this area, earlier seminal studies that have shaped the understanding of childhood eating behaviors within the SEM framework were also considered.

To identify relevant studies, a combination of Medical Subject Headings (MeSH) terms and keywords was employed, incorporating terms such as “childhood eating behaviors”, “familial determinants”, “parental influence”, “social factors”, “peer influence”, “food environment”, and “Social Ecological Model”. Boolean operators were used to refine the search strategy, ensuring a broad yet targeted selection of literature.

Given the narrative nature of this review, the study selection process was guided by a conceptual approach rather than a rigid systematic framework. Studies were included based on their relevance to familial, social, and environmental determinants of childhood eating behaviors, particularly those that aligned with the SEM framework at the individual, interpersonal, community, and policy levels. Preference was given to empirical research that provided insights into caregiver feeding practices, peer influences, school food environments, and the impact of digital media on dietary behaviors. Studies were excluded if they focused exclusively on clinical dietary interventions without addressing broader social or familial determinants. Additionally, reviews, editorials, commentaries, and non-peer-reviewed sources were not considered, ensuring that the synthesis remained rooted in empirical evidence.

Rather than adhering to the structured methodology of a systematic review, this narrative synthesis allowed for a holistic exploration of interconnected influences on childhood eating behaviors. Once the relevant literature was identified, findings were categorized according to the SEM framework, providing a structured yet flexible analysis of how various determinants interact to shape dietary habits. This approach preserves the depth and breadth of discussion characteristic of narrative reviews while enhancing clarity and transparency in the study selection process.

## 3. Results

The key determinants of childhood eating behaviors and corresponding intervention strategies are systematically outlined in Table 1. This table provides a synthesized framework for understanding the multifactorial influences on children’s eating behaviors, encompassing individual, familial, and social-environmental levels. By summarizing critical areas such as parental modeling, the home food environment, peer interactions, screen-viewing behaviors, and school meal programs, Table 1 highlights the complexity of these determinants, and the targeted strategies required to address them. The subsequent sections provide a detailed analysis of these determinants, emphasizing their unique contributions to dietary behaviors and offering evidence-based insights for intervention development.

### 3.1. Food Availability and Accessibility

The relationship between food availability, accessibility, and children’s dietary behaviors is complex and multifaceted, with parents playing a crucial role in shaping their children’s eating habits, particularly in young-age children. Studies have consistently shown that children tend to consume food that is readily available and easily accessible to them [29,30].

Parents are primarily responsible for making food available at home, and their dietary preferences significantly impact the types of food present in the household [8]. This, in turn, affects children’s diet preferences and consumption patterns. Studies have also found that parental modeling, home food availability, and household budgetary constraints are key factors influencing children’s eating behaviors [26,31]. For instance, children whose families cooked dinner at home more than five times per week showed higher consumption of fruits and vegetables and lower consumption of soda [32]. Moreover, the concept of accessibility goes beyond mere availability, encompassing factors such as the convenience of food location and its readiness for consumption [33,34]. Research has demonstrated that children are more likely to eat not only available food but also food that is easily accessible in terms of size and location, as well as foods that are ready to be eaten [29,35].

Interestingly, the neighborhood food environment has less impact on children’s diets than household and parental factors, with the exception of fast food availability. For instance, children with access to fast food within 500 m of their homes were significantly less likely to consume vegetables [36]. This suggests that while the broader food environment plays an important role, the immediate home environment and parental influences are more critical in determining children’s dietary habits. Furthermore, parents are responsible for educating children about healthy and sustainable food choices. A study in urban Poland found that over 80% of parents agreed that they share responsibility in teaching children about the links between food, health, and the environment. However, not all parents are actively engaged in raising their children’s awareness about sustainable behaviors, showing the lack of time or distrust of knowledge in these topics as barriers [36].

To improve children’s diet quality, interventions might need to focus on the home food environment, parental dietary habits, and familial eating practices. For example, in Hispanic children, factors such as the availability of sugar-sweetened beverages at home and family meals while watching television were associated with lower diet quality scores [37]. This emphasizes the need for family-based interventions that address not only food availability and accessibility but also broader eating habits and behaviors. In conclusion, while children tend to consume food that is served and readily available to them, the role of parents in shaping the home food environment is paramount. By considering both the availability and accessibility of foods, along with parental modeling and education about healthy and sustainable choices, interventions can be more effectively directed to promote healthier dietary behaviors among children.

### 3.2. Parents’ Eating Behaviors

The association between parent and child eating behaviors is well-documented and highlights parents’ significant role in shaping their children’s dietary habits. Parents serve as primary role models, influencing children through both direct behaviors, such as the types of foods they offer at home, and indirect behaviors, such as their own eating patterns and attitudes toward food [8]. Studies have shown that children are more likely to adopt healthy eating habits, such as consuming fruits and vegetables when their parents model similar behaviors [26,38].

Parent influence is a significant determinant of children’s dietary habits, often mediated through both direct behaviors and broader socioeconomic contexts. Studies have collectively shown that parental education levels are linked to children’s eating patterns [39]. For example, recent studies have revealed that children with parents who have lower educational attainment tend to consume fewer nutritious foods like processed food and dairy products [40,41]. This suggests that higher parental education, including nutritional knowledge, can greatly enhance their ability to make informed food choices and promote healthier eating habits for their children. Additionally, socioeconomic status can play a significant role in shaping children’s dietary patterns. For instance, children from higher-income families are more likely to exhibit picky eating and fast-food preferences [26,42]. Overall, these findings highlight the importance of parental influence, not only through direct modeling of eating habits but also through broader socioeconomic and educational factors.

These insights underscore the need for comprehensive strategies that consider both individual and environmental factors in promoting healthy eating behaviors among children. Interventions aimed at improving children’s dietary habits may benefit from focusing on educating parents about healthy eating and the importance of physical activity, as well as creating supportive environments that encourage healthy food choices. Studies have shown that interventions focusing on parental modeling and structured mealtime behaviors yield significant improvements in children’s eating habits. For example, programs aimed at enhancing parental engagement during meals—such as involving children in meal preparation and encouraging mindful eating practices—have been associated with better diet quality and reduced risk of obesity [13,43].

### 3.3. The Social Context of Meals

The social context of meals plays a crucial role in shaping children’s eating behaviors. Family mealtimes provide an opportunity for parents to model healthy eating habits and create a positive atmosphere around food. Recent studies have indicated that children who regularly dine with their family members are more likely to consume a balanced diet rich in essential nutrients [44,45,46]. This correlation is supported by various studies demonstrating that the frequency of family meals is positively associated with the intake of healthier food options, such as fruits, vegetables, and whole grains [47,48]. Moreover, the role of companionship at mealtimes extends beyond immediate dietary choices. Engaging in family meals provides a structured environment where positive eating behaviors can be modeled and reinforced. Parents and guardians serve as role models, demonstrating healthy eating practices and fostering an atmosphere conducive to balanced nutrition [49]. This interaction helps inculcate good eating habits in children, which can persist into adulthood. In this note, studies have suggested that regular family meals, particularly those involving parents, may play a critical role in mitigating the risk of obesity, particularly among young children [47,50]. For instance, a recent review by Snuggs and Harvey (2023) showed that, despite the mixed results regarding the effectiveness of interventions, more frequent family meals are consistently associated with improved diet quality, mental health, and academic performance in children and adolescents [46]. Shared mealtimes can strengthen family bonds, improve communication, and provide emotional support, all of which contribute to a child’s overall well-being. This holistic approach underscores the importance of family dynamics in shaping children’s dietary habits and health outcomes [51]. In conclusion, companionship at mealtimes, particularly with family members, plays a pivotal role in influencing children’s dietary choices and overall diet quality. The frequency and quality of family meals are crucial determinants of healthier eating behaviors, which in turn can reduce the prevalence of obesity among children. As such, family-based interventions aimed at increasing the regularity of shared meals should be considered a vital component of public health strategies to combat childhood obesity and promote lifelong healthy eating habits.

### 3.4. Screen Viewing

Screen viewing (such as television watching, computer use, and mobile device usage) has been increasingly associated with changes in eating behaviors and obesity among children in recent years [52]. Recent data have revealed that over one-third of children and adolescents spend more than four hours per day engaged in screen-based activities [53,54]. This extensive screen time is linked to unhealthy eating behaviors and a higher risk of obesity. Increased screen viewing, particularly television, correlates with higher consumption of calorie-dense, nutrient-poor foods, such as sugary drinks and fast food [55,56]. Additionally, food advertisements targeting children further promote unhealthy dietary choices, while mindless eating during screen time leads to increased calorie intake without recognizing satiety cues [57,58].

The sedentary nature of screen viewing also plays a significant role in weight gain. Prolonged screen time reduces physical activity, leading to lower overall energy expenditure and a higher risk of obesity [59]. Additionally, screen use, especially in the evening, is linked to poor sleep quality, which further contributes to weight gain by disrupting hormonal processes that regulate appetite and metabolism [60]. Importantly, the COVID-19 pandemic has significantly altered children’s screen habits, with increased digital engagement due to remote learning, restricted outdoor activities, and greater reliance on electronic devices for entertainment. Studies [61,62,63] have indicated that post-pandemic screen exposure remains higher than pre-pandemic levels, raising concerns about long-term implications for dietary behaviors and sedentary lifestyles. These changes highlight the need for continued monitoring of screen time trends and their impact on eating behaviors.

Interventions targeting screen time reduction have shown promise in improving eating behaviors and reducing the risk of obesity. For example, digital and in-person family-based programs targeting these behaviors have demonstrated positive outcomes such as modest reductions in body mass index and improvements in diet and physical activity levels [64]. Health and education policymakers can enforce stricter regulations on digital marketing of unhealthy foods targeted at children and promote school-based screen time guidelines. Expanding public health campaigns, such as those led by the CDC’s “Screen Time vs. Lean Time” initiative, can further educate parents on setting screen limits and creating tech-free eating spaces at home [65]. Additionally, at the local level, schools and community health programs can implement screen-free initiatives to encourage families to eat without digital distractions. Schools can also integrate structured physical activity breaks and digital literacy education to promote awareness of the link between excessive screen use and unhealthy eating habits. The success of these interventions depends largely on parental involvement and the home environment. Addressing childhood obesity requires a multifaceted approach, including reducing screen viewing, promoting physical activity, and encouraging mindful eating practices.

### 3.5. Eating Out

Eating out or dining outside the home has become an increasingly common practice among families, contributing to significant changes in children’s eating behaviors and a rising risk of obesity. Over time, families are spending more money on dining outside. For example, in 1970, the budget that an average family spent on food was 34%, only to reach 47% by the late in the 1990s [28,66]. Various studies have found a significant association between dining outside the home and the high consumption of dietary fat and energy as compared with dining at home [67,68]. Meals consumed outside the home, particularly in fast food restaurants, are often high in calories, fats, and sugars, while being low in essential nutrients such as fiber, vitamins, and minerals [69]. Frequent consumption of these energy-dense, nutrient-poor meals can lead to excessive calorie intake and weight gain in children.

Additionally, the mode of dining, whether through drive-through fast food options or sit-down restaurants, further influences the nutritional quality of meals and their impact on children’s eating behaviors and weight outcomes. Drive-through dining, for example, has become increasingly prevalent among families due to its quick service and accessibility; however, this dining mode is strongly associated with high calorie food and lack of mindful eating [69,70,71]. In contrast, sit-down dining, whether at casual or full-service restaurants, presents both challenges and opportunities for influencing dietary behavior. Larger portion sizes commonly served in these settings often lead to unintentional overeating, with children consuming significantly more calories compared to meals at home [67,72,73]. Social and environmental factors, such as the presence of others and the availability of calorie-dense options like appetizers and desserts, further promote higher caloric intake through social facilitation [71]. While some establishments offer healthier options like salads, lean proteins, and vegetables, children often gravitate toward high-fat, high-sugar items due to preferences and marketing strategies [74].

The environment of dining out also influences children’s eating behaviors. Research indicates that when eating in fast food or restaurant settings, children are more likely to make unhealthy food choices, such as opting for fried foods, sugary beverages, and desserts, rather than nutrient-rich options like fruits and vegetables [74]. Moreover, the marketing and promotion of unhealthy foods in these settings further encourages poor dietary habits among children [70,75]. These behaviors, combined with a lack of parental control over food preparation and ingredient choices, contribute to the rising rates of childhood obesity associated with frequent eating out.

Interventions aimed at reducing the frequency of eating out or promoting healthier food choices in restaurant settings have shown potential in addressing this issue. Family-based programs that encourage home-cooked meals and teach parents how to make healthier food choices when dining out have been linked to improved diet quality and lower BMI z-scores in children [76]. Additionally, efforts to modify children’s menus in restaurants by offering healthier, smaller portion options have been positively received and could play a role in reducing the risk of obesity in children [77]. Nutrition education programs can partner with restaurants and fast-food chains to promote healthier children’s menus, offering smaller portions and balanced meal options. Initiatives such as “healthy dining partnerships” between municipalities and food service providers can subsidize nutritious menu options, making healthier choices more accessible and affordable for families [78]. At the systemic level, policymakers can implement nutritional labeling regulations, similar to the FDA’s calorie disclosure rules, to ensure that restaurants provide clear dietary information [79]. Additional measures, such as tax incentives for restaurants offering nutritious meals or restrictions on portion sizes for children’s meals, can help reduce calorie-dense, nutrient-poor food consumption [80]. By integrating policy-driven incentives with community-based educational efforts, these interventions can collectively foster healthier eating behaviors when dining outside the home

### 3.6. Feeding Practice

Parental feeding practices play a crucial role in shaping children’s eating behaviors and can significantly influence the risk of obesity. Research shows that parents who practice restrictive feeding, where certain foods are limited or forbidden, may inadvertently promote overeating or unhealthy food choices when those restricted items become available [81]. This restrictive approach often leads children to develop a heightened preference for high-calorie, “forbidden” foods, contributing to excessive calorie intake and weight gain [82]. On the other hand, overly controlling feeding practices can impair a child’s ability to self-regulate their food intake, further increasing the risk of obesity.

Another key factor is parental modeling of eating behaviors. Parents who exhibit healthy eating habits and offer a variety of nutritious foods are more likely to have children who develop healthy dietary patterns [8]. Studies demonstrate that when parents consistently model balanced diets rich in fruits, vegetables, and whole grains, children are more likely to adopt these behaviors [26]. Conversely, parents who frequently consume unhealthy foods, such as sugary snacks and fast food, tend to foster similar eating behaviors in their children [38], which can increase the risk of obesity over time.

Feeding practices that promote autonomy and positive mealtime experiences have shown to be protective against obesity. Practices such as encouraging children to listen to their hunger and satiety cues, allowing them to make their own food choices within a healthy framework, and involving them in meal preparation can foster healthier eating habits and support weight management [13]. Parental involvement and support during meals, without being overly controlling, helps children develop a balanced relationship with food, reducing the likelihood of overeating and obesity [43]. These findings underscore the importance of fostering a positive and balanced feeding environment to promote healthy eating behaviors and reduce obesity risk in children.

### 3.7. Peers’ Eating Behaviors

The eating behaviors of peers can significantly influence children’s eating habits and contribute to the development of obesity. Studies have demonstrated that children tend to mimic the eating behaviors of their peers, particularly in social settings such as school or playgroups [83,84]. When children are exposed to peers who frequently consume unhealthy foods, such as sugary snacks or fast food, they are more likely to adopt similar behaviors, leading to increased consumption of calorie-dense, nutrient-poor foods [85,86]. This peer modeling effect plays a critical role in shaping children’s food preferences and overall dietary patterns, which can contribute to the risk of obesity.

Social dynamics among peers also influence the quantity of food consumed by children. Studies have found that when children eat with peers who consume large portions, they tend to eat more themselves, often exceeding their usual intake [87]. This phenomenon, known as “social facilitation of eating”, can lead to overeating in children, especially in environments where unhealthy foods are readily available. Over time, such patterns of overeating in peer settings may result in excess calorie intake and subsequent weight gain, increasing the likelihood of obesity in children [71].

Interventions aimed at promoting healthy eating behaviors among peer groups can be effective in reducing the risk of obesity [84]. Programs that encourage healthy eating in school settings, such as offering nutritious snacks and meals, as well as fostering positive peer influence, have been shown to improve children’s dietary choices [88]. Additionally, peer-led health education initiatives can be particularly successful, as children are more likely to adopt healthy behaviors when they observe their peers making positive food choices [89]. These findings highlight the powerful role that peer influence plays in shaping children’s eating behaviors and suggest that peer-focused interventions may be an effective strategy for combating childhood obesity.

### 3.8. School Meals

School meals play a pivotal role in shaping children’s eating behaviors and significantly influence both the risk of obesity and broader outcomes such as school attendance and academic performance. Studies have revealed that school meal programs, particularly those meeting national nutritional standards, are associated with healthier eating behaviors in children [90,91,92]. For instance, children who participate in these programs consume balanced meals rich in fruits, vegetables, whole grains, and lean proteins, supporting healthy growth and weight management [93]. These programs are essential in providing nutritious meals to children, especially in low-income populations where access to healthy food may be limited. However, the quality of school meals and the overall school food environment remain critical factors. Children with access to unhealthy snacks and sugary beverages in school settings are more likely to develop poor dietary habits, undermining the benefits of healthier school meals [94,95]. Competitive food options, such as those offered through vending machines or fast food, further exacerbate this issue by encouraging the consumption of calorie-dense, nutrient-poor foods [96]. As a result, children may opt for less healthy options, contributing to excessive calorie intake and weight gain.

Efforts to improve the nutritional quality of school meals have shown promise in reducing obesity risk among children. Interventions such as the Healthy, Hunger-Free Kids Act of 2010 in the United States, which sets stricter nutritional guidelines for school meals, have led to improvements in children’s diet quality and reduced obesity rates [97]. Additionally, programs that educate children about healthy eating, alongside providing nutritious meals, help reinforce positive dietary behaviors [98]. Similarly, programs like BATB emphasize how meal accessibility can positively influence attendance and potentially improve academic outcomes, reinforcing the importance of integrating meal programs with broader educational policies to maximize their impact [92]. These initiatives suggest that improving the school food environment is a critical component in combating childhood obesity and promoting lifelong healthy eating habits.

## 4. Strength, Limitation, and Future Research Considerations

This review has several strengths, including its use of the SEM as a guiding framework, which allows for a comprehensive synthesis of multi-level determinants influencing childhood eating behaviors. The SEM approach ensures a holistic examination of individual, interpersonal, community, and policy-level factors, making it possible to identify interactions between these influences rather than viewing them in isolation. Additionally, this review integrates findings from diverse disciplines, including public health, behavioral science, nutrition, and psychology, offering a broad perspective on the complexity of childhood eating behaviors. By considering various determinants, such as caregiver feeding practices, school environments, socioeconomic factors, and policy interventions, this review provides actionable insights for researchers, educators, and policymakers.

However, this study also has limitations. As a narrative review, it does not adhere to a systematic review methodology, meaning it lacks a formal risk of bias assessment and an exhaustive study selection process. This may introduce selection bias, as the inclusion of studies is based on the authors’ judgment rather than predefined eligibility criteria, potentially overlooking relevant research or disproportionately emphasizing certain findings. Additionally, narrative reviews do not employ standardized appraisal tools to evaluate study quality, making it difficult to assess the reliability and validity of the included evidence. Another limitation is the potential for subjective interpretation of findings, as the synthesis of literature is inherently influenced by the perspectives and expertise of the reviewers. Unlike systematic reviews or meta-analyses, which employ structured data extraction and statistical methods to minimize bias, a narrative review relies on thematic analysis, which may introduce variability in how findings are categorized and interpreted. Furthermore, while this review aims to provide a comprehensive overview of multi-level determinants of childhood eating behaviors, it may not fully capture emerging trends or unpublished research, such as gray literature or ongoing studies that could contribute valuable insights. The exclusion of these sources may lead to publication bias, where only studies with significant or favorable outcomes are considered.

To address these limitations, future research should consider conducting systematic reviews or meta-analyses to provide a more rigorous synthesis of the evidence by applying standardized inclusion criteria, quality appraisal tools, and statistical methods to assess the strength of associations between determinants and childhood eating behaviors. Additionally, scoping reviews may be beneficial to map broader literature trends and identify underexplored areas requiring further empirical investigation. Moreover, future reviews should incorporate gray literature and unpublished studies to reduce publication bias and provide a more comprehensive understanding of the field. Conducting longitudinal and experimental studies could also help establish causal relationships between determinants and eating behaviors, thereby strengthening the evidence base for developing targeted interventions and policies. Despite these limitations, this narrative review provides a critical foundation for advancing research on childhood eating behaviors and serves as a resource for guiding future systematic inquiries and evidence-based policymaking.

## 5. Research Gaps and Future Directions in Childhood Eating Behaviors

This narrative review highlights the complex interplay of familial and social determinants in shaping childhood eating behaviors. While existing research has provided valuable insights into the influence of parental modeling, peer interactions, school food environments, and digital marketing, several gaps remain that warrant further investigation. First, longitudinal studies are needed to better understand the long-term impact of familial and social determinants on dietary behaviors. Most existing studies rely on cross-sectional designs, limiting the ability to establish causal relationships. Future research should explore how early-life eating habits evolve over time and how different influences interact across developmental stages. Second, while digital marketing and screen exposure have been linked to unhealthy dietary choices, intervention-based studies are necessary to determine effective strategies for mitigating these effects. Research should examine the role of media literacy programs, parental controls, and policy regulations in reducing children’s susceptibility to unhealthy food marketing. Third, cultural and socioeconomic factors play a critical role in shaping dietary behaviors, yet much of the existing literature is based on high-income, Western populations. Future studies should focus on underrepresented communities, particularly in low- and middle-income countries, to assess how social and familial determinants influence eating behaviors across diverse cultural contexts. Additionally, while school meal programs have been recognized as an essential component of child nutrition, more research is needed on the effectiveness of school-based interventions that incorporate nutrition education, behavioral strategies, and peer-led initiatives. Understanding how different school policies influence eating behaviors and how to sustain these interventions long-term remains a priority.

Finally, given the emerging role of technology-driven interventions, future research should explore how virtual reality (VR), mobile applications, and digital coaching can be leveraged to improve childhood eating behaviors. Investigating the effectiveness of these tools in real-world settings can provide new opportunities for promoting healthier dietary habits. By addressing these gaps, future studies can contribute to the development of evidence-based, multi-level interventions that support lifelong healthy eating behaviors in children. Integrating findings from diverse disciplines, including public health, psychology, behavioral science, and digital health, will be crucial in designing effective strategies to combat childhood obesity and promote sustainable dietary habits.

## 6. Conclusions

This review highlights the multifaceted nature of childhood eating behaviors, emphasizing the interplay between individual, familial, social, and environmental factors as outlined through the SEM. The findings underscore the significant role of parental modeling, feeding practices, family meal structures, and peer influences in shaping children’s dietary habits. Additionally, broader determinants such as screen time, school meal quality, and food accessibility contribute to variations in dietary behaviors, further moderated by socioeconomic and cultural contexts. These insights reinforce the need for family-centered interventions that prioritize caregiver education, the promotion of responsive feeding practices, and the creation of a supportive home food environment. However, given the complexity of these influences, single-level interventions are insufficient. Instead, multi-level, context-specific strategies that incorporate school-based programs, community initiatives, and policy-level changes are essential for effectively promoting healthy eating habits in children. Policymakers and public health practitioners can prioritize integrated strategies that enhance caregiver education, school-based interventions, and food accessibility improvements to create and promote a sustainable healthier food environment for children. By leveraging multi-sector collaborations and evidence-based policies, it is possible to foster sustainable dietary habits that support long-term child health and obesity prevention.

## Figures and Tables

**Table 1:** A summary of the social ecological perspective on determinants of children’s eating behaviors: influences and interventions.

SEM Level	Determinant	Key Findings	Proposed Interventions
Community	Food Availability and Accessibility	Children tend to consume food that is readily available and accessible at home. Parental food choices, home food availability, and household constraints play key roles in shaping dietary habits.	Enhance home food availability of nutritious options, educate parents on healthy food choices, and promote sustainable eating behaviors.
	Eating Out	Frequent eating out, especially at fast food restaurants, contributes to excessive calorie intake and poor diet quality.	Promote healthier restaurant menu options, nutrition labeling, and policies that encourage home-cooked meals.
Interpersonal	Parents’ Eating Behaviors	Parents serve as role models in shaping children’s dietary habits. Parental education, socioeconomic status, and food choices significantly impact children’s eating behaviors.	Parental education on healthy eating, structured mealtime behaviors, and involvement in meal preparation to reinforce positive dietary habits.
	The Social Context of Meals	Family mealtimes positively influence children’s dietary choices. Regular family meals are associated with higher nutrient intake and lower risk of obesity.	Encourage family meals, implement programs to enhance structured mealtimes, and promote positive parental role modeling.
	Feeding Practice	Parental feeding practices influence children’s eating behaviors. Restrictive feeding may promote overeating, while supportive feeding encourages healthy habits.	Educate parents on feeding practices that support children’s self-regulation and create positive mealtime experiences.
	Peers’ Eating Behaviors	Peer influence affects children’s food choices. Children are likely to adopt the eating behaviors of their friends and social circles.	Develop peer-led health education programs and create school environments that encourage healthy eating habits.
Individual	Screen Viewing	Excessive screen time is linked to poor dietary habits and obesity. Screen exposure promotes unhealthy food choices and increases sedentary behavior.	Implement screen time guidelines, regulate digital food marketing, and promote tech-free meals to reduce mindless eating.
Organizational	School Meals	School meal programs influence children’s nutrition. Nutritional quality and accessibility of school meals impact dietary choices and obesity risk.	Enhance school meal programs with stricter nutritional guidelines, reduce access to unhealthy food options in schools, and integrate nutrition education into curricula.
